# Regulation of Tumor Necrosis Factor-α by Peptide Lv in Bone Marrow Macrophages and Synovium

**DOI:** 10.3389/fmed.2021.702126

**Published:** 2021-07-27

**Authors:** Manabu Mukai, Kentaro Uchida, Tadashi Okubo, Shotaro Takano, Toshihide Matsumoto, Masashi Satoh, Gen Inoue, Masashi Takaso

**Affiliations:** ^1^Department of Orthopaedic Surgery, Kitasato University School of Medicine, Sagamihara, Japan; ^2^Shonan University of Medical Sciences Research Institute, Chigasaki, Japan; ^3^Department of Laboratory Animal Science, Kitasato University School of Medicine, Sagamihara, Japan; ^4^Department of Pathology, Kitasato University School of Medicine, Sagamihara, Japan; ^5^Department of Immunology, Kitasato University School of Medicine, Sagamihara, Japan

**Keywords:** V-set and transmembrane domain-containing 4, peptide Lv, tumor necrosis factor-α, macrophage, synovitis

## Abstract

**Background:** Bone marrow-derived monocytes/macrophages are recruited into synovial tissue, where they contribute to synovial inflammation in osteoarthritis through inflammatory cytokine production. Recent studies have suggested that V-Set and transmembrane domain-containing 4 (VSTM4) and its fragment, peptide Lv, exhibit immunosuppressive activity on T cells and vascular endothelial growth factor (VEGF)-like activity, respectively. Given that evidence suggests that VEGF may play a role in macrophage function, we investigated peptide Lv-mediated regulation of inflammatory cytokines in bone marrow macrophages (BMMs) and synovial inflammation.

**Method:** To investigate the effects of peptide Lv, BMMs were stimulated with vehicle, LPS, or LPS + peptide Lv, and *Tnfa, Il1b, Il6*, and *Ifng* expression were evaluated using quantitative PCR (qPCR). TNF-α and IFN-γ production was measured using ELISA. To examine the effect of peptide Lv deficiency on macrophages and synovitis, *peptide Lv*-deficient mice were generated using genome editing. LPS-induced *Tnfa* and *Ifng* expression and TNF-α and IFN-γ production were evaluated in BMM isolated from wild-type and *peptide Lv*-deficient mice. Additionally, *Tnfa* and *Ifng* expression levels were compared between wild-type and *peptide Lv*-deficient mice before and after knee injury.

**Results:** Peptide Lv suppressed the LPS-mediated elevation in TNF-α and IFN-γ. LPS stimulation significantly increased TNF-α and IFN-γ production in BMM derived from *peptide Lv*-deficient mice compared to wild-type mice. Synovial TNF-α expression in the injured knee was elevated in *peptide Lv*-deficient compared to wild-type mice.

**Conclusion:** Peptide Lv suppressed TNF-α in macrophages and plays a role in synovial inflammation. Thus, peptide Lv may be a useful therapeutic target for synovitis.

## Introduction

The main features of knee osteoarthritis (KOA), a form of degenerative joint disease, are articular cartilage degeneration, osteophytosis, bone remodeling, fibrosis, and synovial hyperplasia (1). Evidence suggests that synovial inflammation may play a role in disease progression and structural changes in KOA (2).

Myeloid cells including macrophages and monocytes contribute to the production of inflammatory cytokines including tumor necrosis factor-α, interleukin-1β, and interleukin-6 in synovitis (3–5). These inflammatory cytokines promote cartilage degradation and pain in the osteoarthritic synovium (4, 5). Several studies have reported that bone marrow-derived monocytes are recruited into synovial tissue, where they contribute to synovial inflammation through inflammatory cytokine production (2, 6, 7). Further, we previously reported that bone marrow-derived myeloid populations were recruited into the synovium in a KOA mouse model (8). Therefore, regulation of macrophage/monocyte-secreted inflammatory cytokines may be an effective therapeutic strategy for treating synovial inflammation.

Recent evidence indicates that the V-Set and transmembrane domain-containing (VSTM) family of proteins plays a variety of roles in the body, including controlling T cell activity, tumor growth, and adipocyte differentiation (9–12). V-Set and transmembrane domain-containing 4 (VSTM4) is a transmembrane protein composed of 320 amino acids (aa). The VSTM4 fragment (human, aa 55–94; mouse aa 55–103), peptide Lv, augments L-type voltage-gated calcium channels and has similar biological actions to vascular endothelial growth factor (VEGF) (13, 14). VEGF is thought to regulate the inflammation process by promoting the polarization of M1 macrophages to M2 macrophages and inhibiting TNF-α-mediated apoptosis (15–17). Given that peptide Lv can activate VEGF signaling, we hypothesized that peptide Lv regulates macrophage activity and plays a role in synovial inflammation.

Here, we examined the role of peptide Lv on inflammatory cytokine expression in macrophages and synovial inflammation.

## Methods

### Animal Ethics

All experimental protocols received approval from the Kitasato University School of Medicine Animal Care Committee (2020-087). The study complied with the ARRIVE guidelines for the reporting of animal experiments. All methods were performed according to the guidelines for the proper conduct of animal experiments by the Science Council of Japan. All experiments were performed a minimum of two times.

### Preparation of Bone Marrow-Derived Macrophages

BMMs grown in monocyte colony-stimulating factor (M-CSF) were established based on a protocol reported previously (18). In brief, bone marrow cells isolated from the femurs of C57BL/6J mice were cultured in α-MEM with 100 ng/ml M-CSF (BioLegend, San Diego, CA, USA). On the fourth day of culture, non-adherent cells were extracted and cultured for an additional 3 days in α-MEM containing fresh M-CSF.

### Impact of Peptide Lv on LPS-Mediated Inflammatory Cytokine Expression

BMMs were isolated from C57BL/6J mice as described above. Synthetic peptide Lv (DSLLAVRWFFAPDGSQEALMVKMTKLRIIQYYGNFSRTANQQRLRLLEE) was purchased from Phoenix Pharmaceuticals (Burlingame, CA, USA). Our pilot studies showed that while 1 μg/ml peptide Lv suppressed *Tnfa* expression, 0.1 μg/ml peptide Lv did not. Therefore, we used 1 μg/ml peptide Lv in this study.

BMMs were exposed to vehicle (α-MEM), 1 μg/ml lipopolysaccharide (LPS) (Enzo Life Sciences Inc., Farmingdale, NY, USA), or 1 μg/ml LPS + 1 μg/ml peptide Lv for 6 and 24 h. Thereafter, total RNA was extracted from BMM and used for cDNA synthesis based on a previously published protocol. The sequences of primers used for qRT-PCR are listed in Table 1. Relative *Tnfa, Il1b, IL6*, and *Ifng* expression was determined using CFX-96® (Bio-Rad, Richmond, CA, USA) and normalized to levels of the housekeeping gene, *Gapdh*. TNF-α and IFN-γ concentration in the supernatant 24 h after treatment was measured using a commercial ELISA kit (BioLegend) according to the manufacturer's protocol.

**Table 1:** Sequences of the primers used in this study.

**Primer**	**Sequence (5^**′**^-3^**′**^)**	**Product size (bp)**
*Tnfa*-F	CTGAACTTCGGGGTGATCGG	122
*Tnfa*-R	GGCTTGTCACTCGAATTTTGAGA	
*Il1b*-F	GCAACTGTTCCTGAACTCAACT	89
*Il1b*-R	ATCTTTTGGGGTCCGTCAACT	
*Il6*-F	CTGCAAGAGACTTCCATCCAG	131
*Il6*-R	AGTGGTATAGACAGGTCTGTTGG	
*Ifng*-F	AGACAATCAGGCCATCAGCAA	134
*Ifng*-R	GGACCCCTGTGGGTTGTTGACC	
*Gapdh*-F	AACTTTGGCATTGTGGAAGG	223
*Gapdh*-R	ACACATTGGGGGTAGGAACA	

### Generation of *peptide lv*-Deficient Mice

To delete the nucleotide sequence encoding *peptide Lv*, we designed two guide RNAs (gRNAa, b) comprising 20-nucleotide sequences targeting exon 2 of the *peptide Lv* gene (5′-CTGTGGTGCTCTCAATGTCA-3′ and 5′-ACCTCTCATTTCCGTCGCCG-3′). The two gRNAs coupled with constant regions of CRISPR RNA (crRNA) and trans-activating crRNA (tracrRNA) were obtained from Thermo Fisher Scientific (Carlsbad, CA, USA). Along with the gRNAs, Cas9 proteins (Integrated DNA Technologies, Coralville, IA, USA) were electroporated into fertilized C57BL/6J zygotes according to a previously published protocol (19). The zygotes were incubated and surviving two-cell-stage embryos were subsequently transferred into the oviducts of pseudo-pregnant female ICR mice.

### Genotyping

Deletion of the target sequence encoding *peptide Lv* was verified by extracting genomic DNA from tissue samples and performing PCR. The specific primer sets for Δ*Lv* mice were F1: 5′-CTTCAGAATTCCAGGTGTTTCC-3′ and R1: 5′-AGTGTCCAAGAGCTGTCCTCAT-3′. DNA was amplified for 35 cycles at 94°C for 20 s, 60°C for 20 s, and 72°C for 20 s using Ex-Taq DNA polymerase (Takara Bio, Shiga, Japan). Mice with the deleted allele showed a band at 413 bp, while wild-type mice showed a band at 777 bp. The Δ*Lv* mouse line was screened based on the genomic DNA sequence. *Peptide Lv* transcripts were obtained by performing PCR using cDNA samples from BMM and synovial tissue taken from wild-type and *peptide Lv*-deficient mice. The following primers were used to detect *peptide Lv* (299 bp) transcripts: F2, 5′-CTATGGGAACTTCAGCCGGAC-3′ and R2, 5′-AGCACACAAGGACAGCGTA-3′.

### Effect of Peptide-Lv Deficiency on LPS-Mediated TNF-α and IFN-γ Production

BMMs were isolated from C57BL/6J mice and *peptide Lv*-deficient mice as described above. BMMs were exposed to vehicle (α-MEM) or 1 μg/ml LPS (Enzo Life Sciences Inc) for 6 and 24 h. *Tnfa* and *Ifng* expression was evaluated using qRT-PCR. TNF-α and IFN-γ concentration in the supernatant 24 h after LPS treatment was evaluated using ELISA, as described above.

### Induction of Synovial Inflammation

Ten-week-old wild-type (C57BL/6J) and *peptide Lv*-deficient mice were kept in a semibarrier system at 23°C ± 2°C and 55 ± 10% humidity under a 12-h light/dark cycle in Nippon Charles River Laboratories (Kanagawa, Japan) for the duration of the study. Synovial inflammation was induced by performing medial parapatellar arthrotomy in one knee of anesthetized wild-type and *peptide Lv*-deficient mice (20). The knee joint was extended and the patella was dislocated laterally. After completely flexing the knee joint again, the rectus femoris was sutured on the lateral side, followed by the skin. One week after injury, synovial specimens were harvested and homogenized with Trizol (*n* = 5). Total RNA extraction, cDNA synthesis, and qPCR for *Tnfa* and *Ifng* were performed as described above.

### Statistical Analysis

Bonferroni multiple comparisons test with one-way analysis of variance was used to determine differences among the vehicle, LPS, and LPS + Peptide Lv treatment groups at each time point. Following a Kolmogorov–Smirnov test, a *t*-test or Mann–Whitney *U*-test was used to compare differences between means (SD) from wild-type and *peptide Lv*-deficient mice. All statistical analyses were performed using SPSS version 19.0 (IBM Corp., Chicago, IL, USA). *P* < 0.05 was considered statistically significant.

## Results

### Effect of Peptide Lv on LPS-Mediated Inflammatory Cytokine Expression in BMM

**Figure 1:**
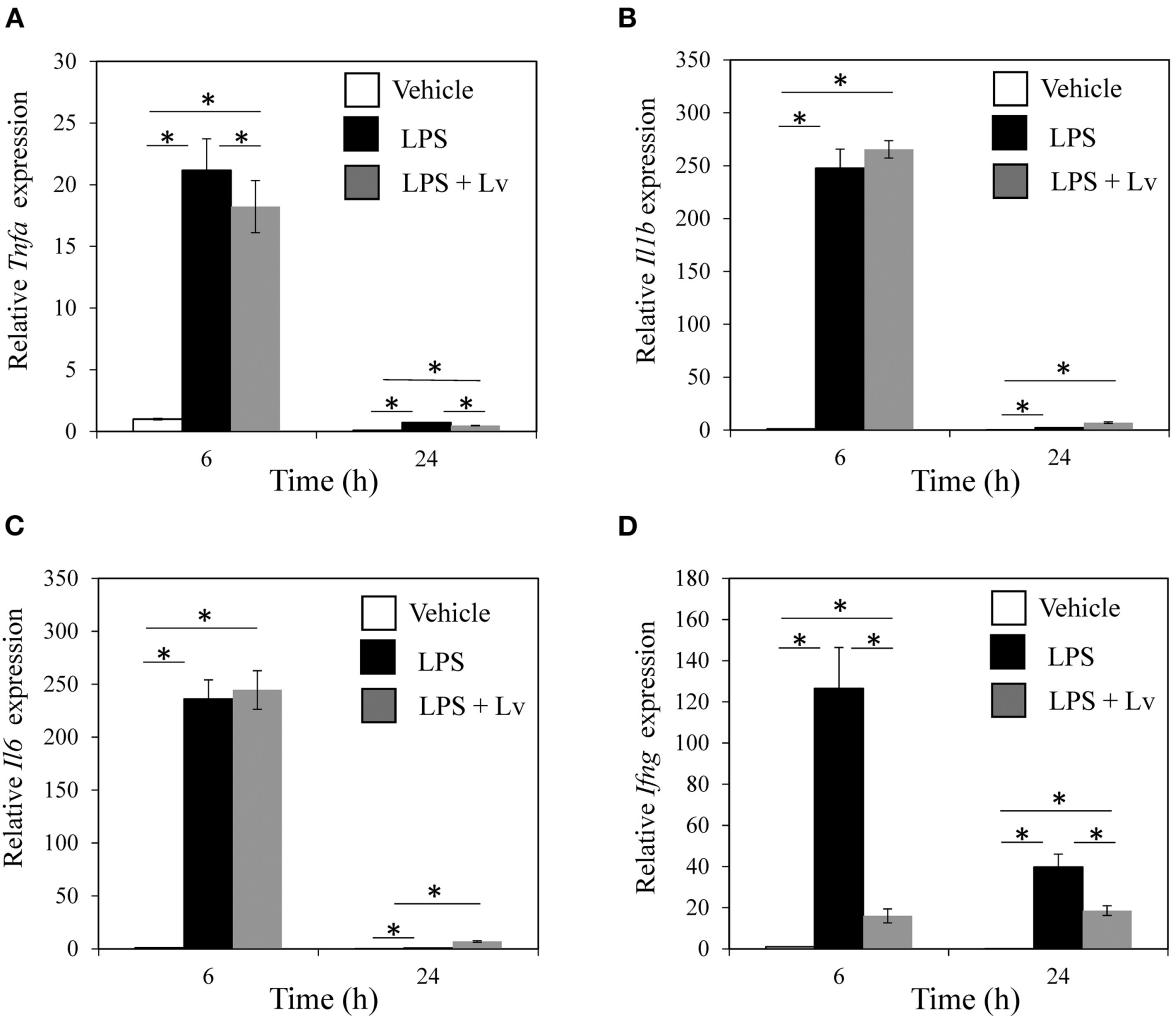
Effect of peptide Lv on LPS-mediated inflammatory cytokine production. **(A)** *Tnfa*, **(B)** *Il1b*, **(C)** *Il6*, and **(D)** *Ifng* expression levels in vehicle-, LPS-, and LPS + peptide Lv (LPS + Lv)-treated BMM at 6 and 24 h. Relative expression was determined based on the expression in vehicle-treated BMM at 6 h. **(B)** TNF-α protein levels in the culture supernatant of vehicle-, LPS-, and LPS + peptide Lv (LPS + Lv)-treated BMM. Values are mean ± SE (*n* = 8). *p < 0.05.

**Figure 2:**
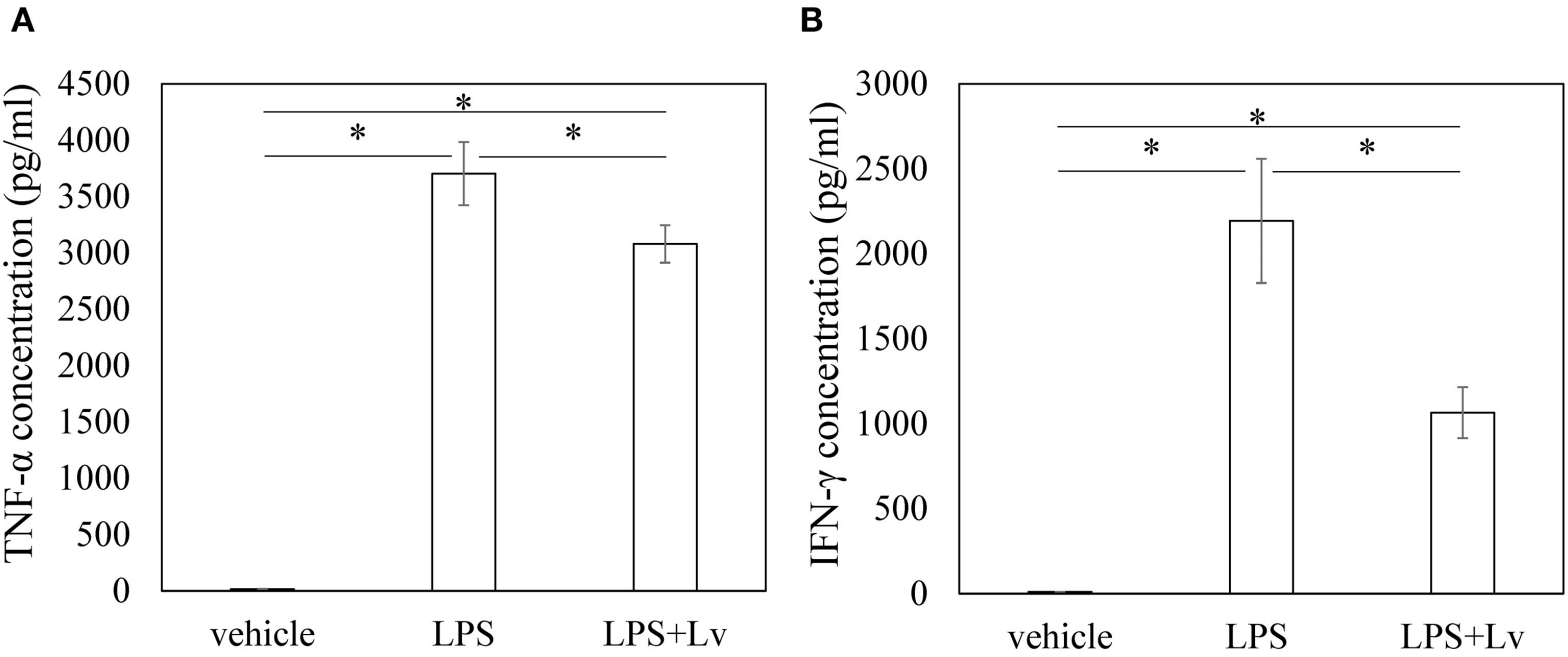
Effect of peptide Lv on LPS-mediated TNF-α and IFN-γ production. **(A)** TNF-α and **(B)** IFN-γ protein levels in the culture supernatant of vehicle-, LPS-, and LPS + peptide Lv (LPS+Lv)-treated BMM. Values are mean ± SE (*n* = 8). *p < 0.05.

We studied the effect of peptide Lv on BMM. Exposure to LPS caused a significant rise in *Tnfa, Il1b, Il6*, and *Ifng* at 6 h (*Tnfa, p* = 0.004; *Il1b, p* < 0.001; *Il6, p* < 0.001; *Ifng, p* = 0.010; Figures 1A–D) and 24 h (*Tnfa, p* = 0.009; *Il1b, p* = 0.001; *Il6, p* < 0.001; *Ifng, p* = 0.0109). Peptide Lv suppressed the LPS-mediated elevation in *Tnfa* and *Ifng* expression at 6 h (*Tnfa, p* = 0.015; *Ifng, p* = 0.011) and 24 h (*Tnfa, p* = 0.002; *Ifng, p* = 0.049). In contrast, there were no changes in *Il1b* or *Il6* expression in the presence of peptide Lv. LPS significantly increased TNF-α and IFN-γ protein concentrations in the supernatant (TNF-α, *p* < 0.001; IFN-γ, *p* = 0.012; Figures 2A,B). These elevated TNF-α and IFN-γ protein concentrations were significantly reduced in the presence of peptide Lv (TNF-α, *p* = 0.029; IFN-γ, *p* = 0.044; Figures 2A,B).

### Effect of Peptide Lv Deficiency on TNF-α Expression

**Figure 3:**
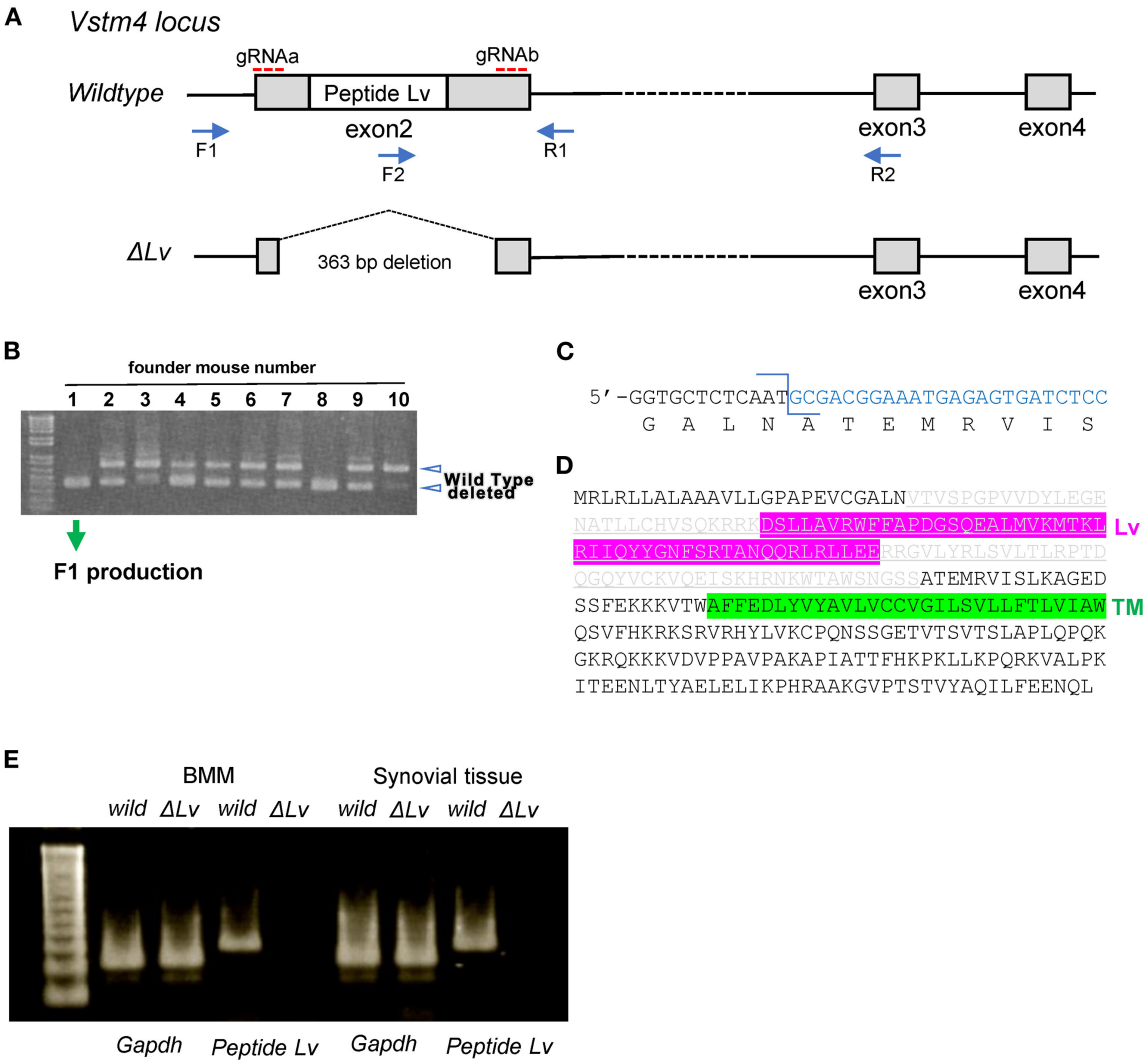
Generation of *peptide Lv*-deficient mice. **(A)** A map of the mouse *Vstm4* locus showing the target regions of the gRNAs and primers used for polymerase chain reaction (PCR). **(B)** Results of genotyping using PCR to screen for *peptide Lv*-deficient founder mice. **(C)** The sequence at the junction of the deleted genes. **(D)** The predicted amino acid sequence of deleted peptide Lv in mouse line No. 1M1. Gray letters are the deleted amino acid sequences. **(E)** Results of RT-PCR to confirm the depletion of Lv.

Given our finding that peptide Lv diminished LPS-mediated TNF-α and IFN-γ production in BMM, we genetically deleted *peptide Lv* to investigate its role in macrophages and synovitis. We designed two gRNAs to delete exon 2 from the *peptide Lv* gene in mice using CRISPR/Cas9 (Figure 3A). *Peptide Lv*-deficient mouse founders (Figure 3B) were crossed with C57BL/6J mice to generate F1 progeny. Genotyping confirmed that all F1 mice were missing exon 2 of the *peptide Lv* gene (Figure 3C). The predicted amino acid sequence of the deleted peptide Lv portion of the Vstm4 protein is shown in Figure 3D. RT-PCR confirmed that *peptide Lv* was successfully deleted in BMM and synovial tissue was taken from a *peptide Lv*-deficient mouse (Figure 3E).

**Figure 4:**
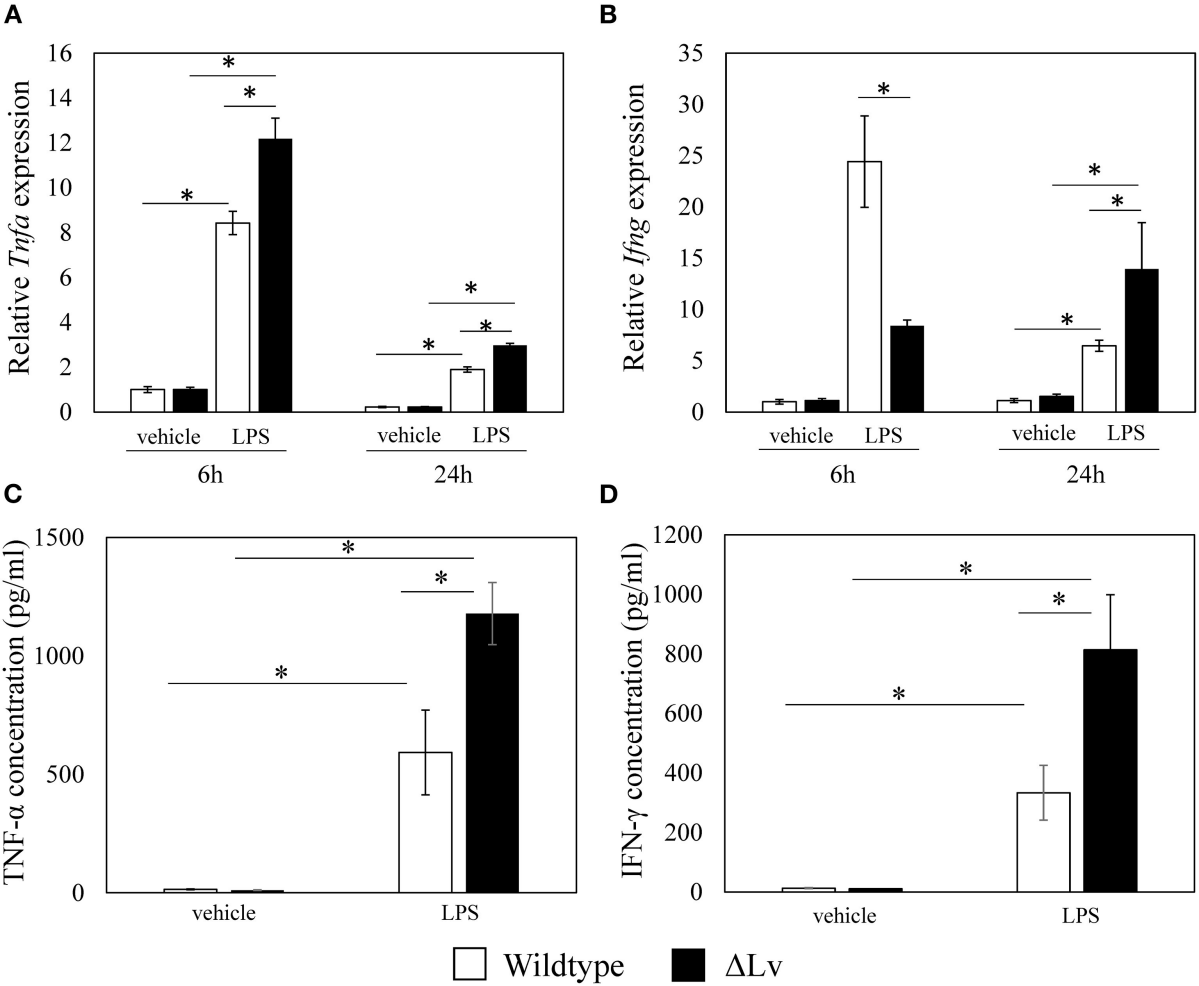
Effect of peptide Lv deficiency on LPS-mediated TNF-α and IFN-γ production. **(A)** *Tnfa* and **(B)** *Ifng* expression levels in vehicle- and LPS-treated BMM obtained from wild-type and *peptide Lv-*deficient (ΔLv) mice. Relative *Tnfa* expression was determined based on the expression in vehicle-treated BMM. **(C)** TNF-α and **(D)** IFN-γ protein levels in the culture supernatant of vehicle- and LPS-treated BMM obtained from wild-type and ΔLv mice. Values are mean ± SE (*n* = 8). *p < 0.05.

There were no differences in *Tnfa* and *Ifng* expression between vehicle-stimulated BMM derived from wild-type and *peptide Lv*-deficient mice at 6 h (*Tnfa, p* = 0.917; *Ifna, p* = 0.602; Figures 4A,B) or 24 h (*Tnfa, p* = 0.917; *Ifna, p* = 0.175; Figures 4A,B). In contrast, stimulation of BMM with LPS significantly increased *Tnfa* expression in BMM derived from *peptide Lv*-deficient mice compared to wild-type mice (6 h, *p* = 0.009; 24 h, *p* = 0.009, respectively; Figure 4A). Consistent with our qRT-PCR results, TNF-α protein levels rose in BMM derived from *peptide Lv*-deficient mice compared to wild-type mice (*p* = 0.001; Figure 4C). While LPS stimulation significantly increased *Ifng* expression in BMM derived from wild-type mice compared to *peptide Lv*-deficient mice at 6 h (*p* = 0.009; Figure 4B), it significantly increased *Ifng* expression in BMM derived from *peptide Lv*-deficient mice compared to wild-type mice at 24 h (*p* = 0.047; Figure 4B). IFN-γ protein levels increased in BMM derived from *peptide Lv*-deficient mice compared to wild-type mice (*p* = 0.028; Figure 4D).

### Effect of Peptide Lv Deficiency on *Tnfa* and *Ifng* Expression During Synovial Inflammation

**Figure 5:**
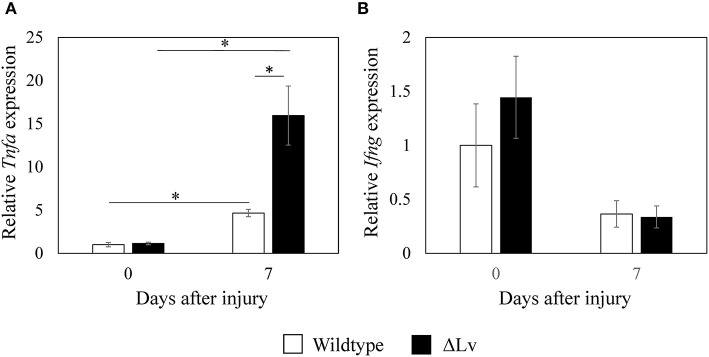
Effect of peptide Lv deficiency on *Tnfa* and *Ifng* expression in synovium. **(A)** *Tnfa* and **(B)** *Ifng* expression in synovial tissue obtained from wild-type and *peptide Lv-*deficient (ΔLv) mice with synovitis. Values are mean ± SE (*n* = 5). *p < 0.05.

*Tnfa* expression levels in the synovium of non-injured wild-type mice were similar to those in non-injured *peptide Lv*-deficient mice (*p* = 0.713; Figure 5A). Synovial *Tnfa* expression increased after injury in both wild-type and *peptide Lv*-deficient mice compared to the non-injured synovium (*p* = 0.009 and *p* = 0.009, respectively). However, synovial *Tnfa* expression after injury was markedly higher in *peptide Lv*-deficient mice than wild-type mice (*p* = 0.011; Figure 5A). No significant differences were observed in *Ifng* expression between wild-type and *peptide Lv*-deficient mice before or after injury (day 0, *p* = 0.347; day 7, *p* = 1.000; Figure 5B).

## Discussion

Previous studies have reported that the VSTM family of proteins suppresses T cells and that recombinant VSTM proteins inhibit T cell function *in vitro*. Recombinant VSTM4 significantly reduces the production of inflammatory cytokines including IFN-γ, IL-2, and IL-17 by T cells (12). Here, synthetic peptide Lv inhibited LPS-mediated TNF-α and IFN-γ production, while peptide Lv deficiency increased TNF-α and IFN-γ production in LPS-stimulated BMM. Our results suggest that peptide Lv may impart anti-inflammatory action on macrophages.

Peptide Lv can interact with the VEGF receptor and trigger downstream signaling in cardiomyocytes (13). Several studies have reported the protective effects of VEGF on inflammatory stimuli (15, 21). VEGF inhibited LPS-mediated apoptosis in endothelial cells (15) and suppressed LPS-mediated M1 polarization in a human monocyte cell line (21). While the mechanism by which peptide Lv regulates TNF-α in our study remains unclear, it is possible that it may involve the VEGF-like actions of peptide Lv.

Studies have shown that TNF-α also regulates the production of pain molecules such as nerve growth factor, calcitonin gene-related peptide, and cycloxgenase-2 (5). In the present study, *Tnfa* expression levels were increased in the synovium of *peptide Lv*-deficient mice. TNF-α is a major molecular component of and drug target for OA (22–25). A TNF-α inhibitor has been shown to improve synovitis and pain in a patient with inflammatory KOA (23). Moreover, intra-articular injections of an anti-TNF-α antibody in a recent pilot study significantly improved KOA symptoms and progression (22, 24). Given that peptide Lv suppresses TNF-α expression, peptide Lv may be a useful therapeutic target for OA.

IFN-γ is primarily produced by T cells and natural killer cells and contributes to macrophage activation (26). However, previous studies have showed that macrophages also produce IFN-γ and are stimulated by LPS (27, 28). Given that a fusion protein containing the extracellular domain of VSTM4 fused to Fc (VSTM4-Fc) was shown to inhibit IFN-γ production in human T cells (12), we evaluated the effect of the VSTM4 fragment on IFN-γ expression in macrophages. We found that peptide Lv suppressed IFN-γ production, while peptide Lv deficiency increased IFN-γ production in macrophages. A previous study of bacterial infection reported that macrophage-derived IFN-γ can activate macrophages via an autocrine/paracrine pathway (29). Together with previous studies, our results suggest that peptide Lv may suppress macrophage activation *via* IFN-γ production. However, in our study, synovial *Ifng* expression did not increase after injury in either wild-type or *peptide Lv*-deficient mice. Therefore, the role that peptide Lv plays in IFN-γ expression during synovial inflammation remains unclear. Further investigation is needed.

Several limitations warrant mention. First, we only studied the effects of a single dose of peptide Lv in the *in vitro* study. Second, the effect of peptide Lv on TNF-α was examined in BMM. These data cannot be directly extrapolated to synovial macrophages. Further investigation using synovial macrophages is needed. Third, the mechanism by which peptide Lv affects TNF-α in macrophages remains unclear. Finally, although synovial *Tnfa* expression was elevated in *peptide Lv*-deficient mice, it remains unclear whether this outcome is due to the effect of macrophages.

## Conclusion

Macrophages express VSTM4 and its fragment, peptide Lv. Peptide Lv suppressed TNF-α and IFN-γ production in macrophages, while peptide Lv deficiency increased *Tnfa* expression in the inflamed synovium. Peptide Lv may thus be a therapeutic target for synovial inflammation.

## Data Availability Statement

The original contributions presented in the study are included in the article/supplementary material, further inquiries can be directed to the corresponding author/s.

## Ethics Statement

The animal study was reviewed and approved by All experimental protocols received approval from the Kitasato University School of Medicine Animal Care Committee (2020-087).

## Author Contributions

MM, KU, and MT designed the experiments. MM, KU, TO, ST, TM, MS, and GI collected the data. MM, ST, TM, MS, and GI performed data analysis and interpretation. MM and KU wrote the manuscript. All authors have read the manuscript and provided intellectual content.

## Conflict of Interest

The authors declare that the research was conducted in the absence of any commercial or financial relationships that could be construed as a potential conflict of interest.

## Publisher's Note

All claims expressed in this article are solely those of the authors and do not necessarily represent those of their affiliated organizations, or those of the publisher, the editors and the reviewers. Any product that may be evaluated in this article, or claim that may be made by its manufacturer, is not guaranteed or endorsed by the publisher.

## Abbreviations

OA: osteoarthritis
VSTM4: V-Set and transmembrane domain-containing 4
VEGF: vascular endothelial growth factor
BMM: bone marrow macrophages
LPS: lipopolysaccharide
TNF-α: tumor necrosis factor-α
IL-1β: interleukin-1β
IL-6: interleukin-6
IFN-γ: interferon-γ.
